# CCR7 Receptor Expression in Mono-MAC-1 Cells: Modulation by Liver X Receptor *α* Activation and Prostaglandin E_**2**_

**DOI:** 10.1155/2015/201571

**Published:** 2015-12-06

**Authors:** Bérengère Tanné, Stéphane Bernier, Nancy Dumais

**Affiliations:** Département de Biologie, Faculté des Sciences, Université de Sherbrooke, Sherbrooke, QC, Canada J1K 2R1

## Abstract

Cell migration via chemokine receptor CCR7 expression is an essential function of the immune system. We previously showed that prostaglandin E_2_ (PGE_2_), an important immunomodulatory molecule, increases CCR7 expression and function in monocytes. Here, we explore the role of the liver X receptor *α* (LXR*α*) activation on CCR7 expression in Mono-Mac-1 (MM-1) cells in the presence of PGE_2_. To do this, MM-1 cells were stimulated with the LXR*α* synthetic agonist T0901317 in the presence or absence of PGE_2_.* CCR7* mRNA transcription was measured using quantitative RT-PCR and protein expression was examined using flow cytometry. CCR7 function was analyzed using migration assays in response to CCL19/CCL21, which are natural ligands for CCR7. Our results show that agonist-mediated activation of LXR*α* in the presence of PGE_2_ increases CCR7 mRNA transcription and MM-1 cell migratory capacity in response to CCL19/21. In addition, our results demonstrate that engagement of the E-prostanoids 2 and 4 (EP_2_/EP_4_) receptors present on MM-1 cells is responsible for the observed increase in* CCR7* mRNA expression and function during LXR*α* activation. Examination of monocyte migration in response to lipid derivatives such as PGE_2_ and oxysterols that are produced at sites of chronic inflammation would contribute to understanding the excessive monocyte migration that characterizes atherosclerosis.

## 1. Introduction

Inflammatory monocytes are rapidly recruited to sites of inflammation, but their excessive and/or prolonged recruitment hinders the resolution of inflammation and is a hallmark of numerous diseases. Chemokines CCL19 and CCL21, which are important for cellular migration, are expressed by lymphatic endothelia as well as within lymph nodes by stromal cells, endothelial cells, and dendritic cells (DCs) [1–4]. These chemokines are the natural ligands of CCR7, which is expressed in DCs [5], T and B cells [6], and monocytes [7]. Mice deficient in CCL19, CCL21, or CCR7 demonstrate defective DC trafficking and altered immune responses [8, 9].

PGE_2_ modulates immune responses both* in vitro* and* in vivo* [10]. A marked increase in PGE_2_ production (as high as 10^−4^ M) is generated in response to a variety of immunological stimuli and infections with different pathogens (reviewed in [11]). The immunomodulatory molecule PGE_2_ appears to have a dual role in DC migration by regulating CCR7 expression and activity. Maturation-induced upregulation of CCR7 surface expression is not sufficient for monocyte-derived DCs (MoDCs) to migrate toward CCL19 and CCL21 [12, 13]. Indeed, MoDC migration toward CCL19 and CCL21 was readily observed upon maturation in the presence of the proinflammatory mediator PGE_2_. However, PGE_2_ did not alter expression levels of CCR7 on mature DCs [12, 13]. In human monocytes, PGE_2_ affects* CCR7* mRNA expression and function [6, 14].

In macrophages as well as in DCs, oxysterol-mediated activation of the nuclear liver X receptor (LXR) has been shown to modulate innate immunity and tumor growth (reviewed in [15]). LXR*β* is expressed ubiquitously, whereas LXR*α* is expressed in the liver, adipose tissue, adrenal glands, intestine, lungs, and cells of the myelomonocytic lineage [16]. Interestingly, it has been demonstrated that LXR-dependent effects in DCs regulate CCR7-dependent migration. Indeed, LXR*α* and LXR*β*, which are oxysterol-activated transcription factors, prevent TLR-induced CCR7 upregulation in MoDCs [17] and interfere with CCR7 expression on mature DCs, resulting in a dampened antitumor immune response [18]. Moreover, a recent study showed that PGE_2_ interferes with LXR activation, downregulates LXR*α* expression, and rescues the migratory ability of DCs to migrate toward CCR7 ligands [19]. Therefore, because lipid derivatives such as oxysterols and prostaglandins are important in DC migration, we examined whether PGE_2_ and LXR activation can modify CCR7-dependent migration of human monocytes.

Our results show that PGE_2_ and synthetic LXR*α* ligand, T0901317, strongly increase MM-1 cell migratory capacity in response to CCL19/21. Examination of monocyte migration in response to lipid derivatives, produced during chronic inflammation, would contribute to understanding the excessive monocyte migration that characterizes atherosclerosis.

## 2. Materials and Methods

### 2.1. Reagents

Prostaglandin E_2_ and the LXR agonist T0901317 were purchased from Sigma-Aldrich (St. Louis, MO, USA). The MEK kinase inhibitor PD98059 was purchased from Enzo Life Sciences (Farmingdale, NY, USA). The EP agonists butaprost, 11-deoxy-PGE_1_, and 17-phenyl-trinor-PGE_2_, as well as the phosphatidylinositol 3-kinase (PI3K) inhibitor LY294002 and PKA inhibitor H-89 were obtained from Cayman Chemical (Ann Arbor, MI, USA). The blocking antibody against human CCR7 and chemokines CCL19/CCL21 were purchased from R&D Systems (Minneapolis, MN, USA).

### 2.2. Cell Culture

Mono-Mac-1 cells (MM-1; ACC 252), an acute peripheral monoblastic leukemia derived cell line (German Collection of Microorganisms and Cell Cultures, Braunschweig, Germany), were cultured in RPMI 1640 media (Sigma-Aldrich) supplemented with 10% heat-inactivated fetal bovine serum (FBS), nonessential amino acids (NEAA), 1 mM sodium pyruvate, 100 I.U. penicillin G, and 100 *μ*g/mL streptomycin (all were obtained from Wisent, St-Bruno, QC, Canada).

### 2.3. Blood Monocyte Isolation

Total blood mononuclear cells were isolated from the blood of healthy donors using lymphocyte separation Medium 1077 (Sigma) and washed twice in Hank's balanced salt solution (Wisent). Cells were cultured in RPMI 1640 medium, 20% heat-inactivated FBS, and 10% heat-inactivated human serum for 2 h before use. Monocytes were washed from nonadherent cells with phosphate-buffered saline (PBS). Monocytes were then enriched from peripheral blood mononuclear cells using the MACS Monocyte Isolation Kit II and MACS LS Columns (Miltenyi Biotec, Auburn, CA, USA), yielding an average purity of 98%. The purity was assessed by flow cytometric analyses as recommend by the manufacturer, and isolated monocytes were fluorescently stained with CD14-FITC and anti-Biotin-PE that labeled nonmonocytes. Blood monocytes were stimulated for the indicated times using 1 *μ*M PGE_2_ and/or 1 *μ*M T0901317.

### 2.4. Real-Time Quantitative Polymerase Chain Reaction (qPCR) Analyses

MM-1 cells were incubated with stimulants for the indicated times, and total RNA was extracted using Nucleospin RNA columns (Macherey-Nagel, BioLynx, Brockville, ON, Canada) according to the manufacturer's instructions. RNA (2 *μ*g) was reverse-transcribed into cDNA in the presence of 0.5 *μ*g of oligonucleotide d(T)_15_, 200 units of M-MLV RT (Promega, Madison, WI, USA), and 250 *μ*M deoxyribonucleotide triphosphates (dNTPs) at 42°C for 1 h. Amplification of human* NR1H3* (*LXRα*),* CCR7*, and* ABCG1* was performed using the SYBR Green I nucleic acid gel stain (Invitrogen, Burlington, ON, Canada) on a CFX Connect Real Time System (BIO-RAD, Mississauga, ON, Canada). Results were analyzed using the software BIO-RAD CFX Manager. qPCR reactions contained 0.25 *μ*M forward and reverse primers (Table 1), 0.1 mM dNTPs, 2 mM or 3.5 mM MgCl_2_, and 1.25 units of Omni Klentaq (Enzymatics, Beverly, MA, USA). In each reaction, we used 8 *μ*L of a 1 : 2 dilution of each cDNA (dilutions were performed using molecular grade sterile water (Wisent)). PCR cycling conditions consisted of an initial denaturation at 95°C for 3 minutes and 40 cycles of 95°C for 10 sec, 60°C for 40 sec, 72°C for 40 sec, and a melting curve at 95°C for 10 sec, 65°C for 5 sec, and 95°C for 5 sec.

### 2.5. Chemotaxis Assays

MM-1 cell chemotaxis was measured by migration through a polycarbonate filter (5 *μ*m pore size) in a 96-well transwell chambers (Millipore, Nepean, ON, Canada). The lower chamber contained either 150 *μ*L of a 300 ng/mL dilution of chemokine CCL19/CCL21 in RPMI 1640 media without FBS, NEAA, and sodium pyruvate, but complemented with 0.25% BSA, or media alone as a spontaneous migration control. The upper chamber contained 2.5 × 10^5^ cells in 75 *μ*L of medium. Chambers containing MM-1 cells were incubated for 4 h at 37°C. An aliquot (150 *μ*L) of cells that migrated to the bottom chamber was mixed with 1x PBS (150 *μ*L) and counted using a BD FACSCalibur flow cytometer (BD Biosciences, San Jose, CA, USA) by acquiring events for a fixed period of 60 seconds using CellQuest Software (BD Biosciences). The percentage of migrated cells was calculated as follows: the number of migrated cells in response to media only was subtracted from the number of migrated cells in response to CCL19/CCL21. This number was normalized to the total input of cells. Each experiment was performed in triplicate and repeated at least three times.

### 2.6. Flow Cytometry Analyses

MM-1 cells were collected and washed twice with PBS supplemented with 3% bovine serum albumin (BSA). Fc receptors were blocked for 15 minutes at room temperature using 100 *μ*L of human serum (diluted 1 : 5 in 1x PBS) for 1 × 10^6^ cells. Cells were then washed twice in PBS plus 3% BSA. Cells were labeled with an anti-CCR7 antibody conjugated with allophycocyanin (APC), or corresponding isotypes as a negative control, for 45 minutes on ice in the dark. Cells were then washed twice with PBS plus 3% BSA and centrifuged 5 minutes at 10 g at 4°C. For intracellular experiments, cells were collected and washed with buffer (1x PBS plus 1% BSA and 0.02% sodium azide). Fc receptors were blocked using 100 *μ*L human serum for 15 minutes at room temperature and washed with wash buffer. Cells were fixed with 200 *μ*L 4% paraformaldehyde for 15 minutes at 4°C and again washed with wash buffer. Cells were permeabilized by adding 200 *μ*L of 1% saponin to the wash buffer and then incubated for 45 minutes on ice with anti-CCR7 coupled with APC (Abcam, San Francisco, CA, USA) or the corresponding isotype controls. Cells were then washed twice with wash buffer. Fluorescence was read using a BD FACSCalibur flow cytometer (BD Biosciences) and results were analyzed using CellQuest software (BD Biosciences).

### 2.7. Statistical Analyses

Each experiment was performed at least three times. Statistically significant differences between experimental groups were evaluated using paired *t*-tests and *p* < 0.05 was considered statistically significant. Computations were performed using GraphPad PRISM version 6.0 statistical software (GraphPad, San Diego, CA, USA).

## 3. Results

### 3.1. PGE_2_ and LXR*α* Activation Upregulate CCR7 mRNA Production and Function without Affecting CCR7 Surface Expression in MM-1 Cells

**Figure 1:**
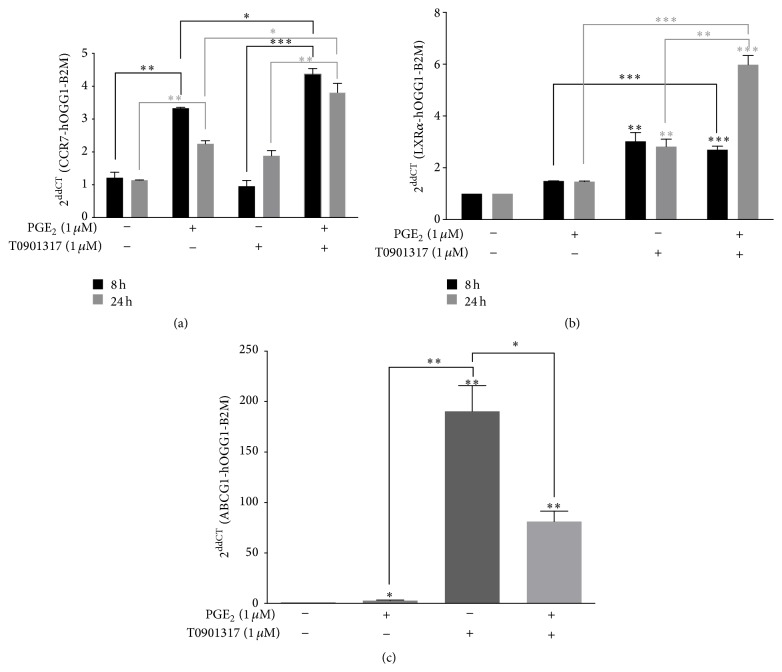
Effects of PGE_2_ and T0901317 on* CCR7*,* ABCG1*, and* LXRα* transcription in MM-1 cells. MM-1 cells were stimulated for 8 h and 24 h with 1 *μ*M PGE_2_ and 1 *μ*M T0901317. Total RNA was extracted and examined using real-time quantitative PCR to detect* CCR7* (a),* ABCG1* (b), and* LXRα* (c) mRNA levels. Data represent mean ± standard deviation (SD) of three independent experiments. ^*∗*^ *p* < 0.05, ^*∗∗*^ *p* < 0.01, and ^*∗∗∗*^ *p* < 0.001.

MM-1 is a human cell line with the properties of blood monocytes that can be used as a model system to study monocytic functions* in vitro* [20]. We first used real-time qPCR to examine whether PGE_2_ and LXR*α* activation could modulate* CCR7* transcription in MM-1 cells. MM-1 cells were treated with 1 *μ*M PGE_2_ and 1 *μ*M T0901317, a synthetic LXR*α* agonist, for 8 or 24 h (Figure 1(a)). As previously observed, PGE_2_ induces* CCR7* mRNA expression with a maximal effect after stimulation for 8 h [7]. Treatment of MM-1 cells with T0901317 alone does not modify the expression of* CCR7* mRNA. Interestingly, we found that cells treated with a combination of PGE_2_ and T0901317 showed significantly upregulated* CCR7* mRNA transcription.

Next, we determined whether MM-1 cells can autoregulate LXR*α* expression as previously demonstrated in macrophages [21].* LXRα* transcripts were measured using real-time qPCR following stimulation with 1 *μ*M PGE_2_ and 1 *μ*M T0901317 (Figure 1(b)). We observed an increase in* LXRα* levels following treatment with PGE_2_ or T0901317 alone. mRNA production plateaued after 8 h of stimulation with PGE_2_ or T0901317 alone. However, MM-1 cells that were stimulated with a combination of PGE_2_ and T0901317 showed increased levels of* LXRα* mRNA after 24 h compared to cells stimulated with PGE_2_ or T0901317 alone.

We also investigated whether PGE_2_ in combination with T0901317 affects the expression of one LXR*α* target gene,* ABCG1* (Figure 1(c)). As expected,* ABCG1* mRNA levels were augmented when cells were treated with T0901317 alone. PGE_2_ had no effect on* ABCG1* transcription. However, MM-1 cells treated with a combination of PGE_2_ and T0901317 showed significantly reduced levels of* ABCG1* transcription.

**Figure 2:**
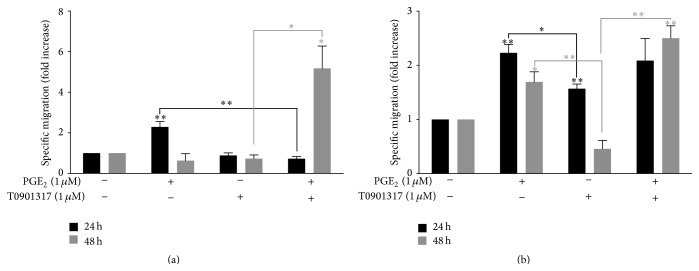
PGE_2_ alone and in combination with T0901317 induces functional CCR7-specific migration of MM-1 cells. Chemotaxis assays in response to 300 ng/mL CCL19 (a) or CCL21 (b) were performed in MM-1 cells stimulated for 24 h (filled black) or 48 h (filled gray) with 1 *μ*M PGE_2_ and 1 *μ*M T0901317. The mean number of spontaneously migrating cells (that migrated to media alone) was subtracted from the number of cells that migrated in response to CCL19 or CCL21. Data represent mean ± SD of three independent experiments. ^*∗*^ *p* < 0.05, ^*∗∗*^ *p* < 0.01.

**Figure 3:**
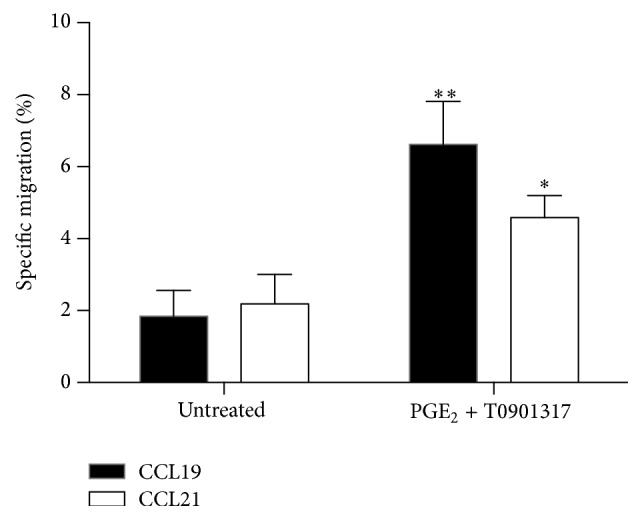
Freshly isolated monocytes migrate toward the CCR7-specific ligands CCL19 and CCL21. Chemotaxis assays using 300 ng/mL CCL19 and CCL21 were performed using human monocytes incubated in the presence or absence of 1 *μ*M PGE_2_ and 1 *μ*M T0901317 for 48 h. Data represent the means of three different experiments. Data represent mean ± SD of three independent experiments. ^*∗*^ *p* < 0.05, ^*∗∗*^ *p* < 0.01 compared to untreated cells.

We next established whether increased mRNA production correlates with CCR7 receptor function in MM-1 cells. MM-1 cells were treated with the stimulants for 24 or 48 h before migration through polycarbonate filters (5 *μ*m pore size) for 4 h. The chemotaxis assay results showed that PGE_2_ increases MM-1 cell migration to both CCR7 natural ligands CCL19 (Figure 2(a)) and CCL21 (Figure 2(b)) after treatment for 24 h. Although the migratory capacity of MM-1 cells is sustained in response to CCL21 after 48 h, migration in response to CCL19 appears transient. The LXR agonist alone did not affect the migratory capacity of MM-1 cells in response to CCL19 whereas 24 h treatment with T0901317 significantly increased migration in response to CCL21. In contrast, migration of MM-1 cells treated with PGE_2_ and T0901317 for 48 h was increased compared to untreated cells or cells treated with PGE_2_ alone. For all chemotaxis assays, we confirmed the specificity of migration by incubating PGE_2_- and T0901317-stimulated cells with a blocking antibody against human CCR7 for 10 min prior to migration assays. Blockade of CCR7 at the MM-1 cell surface completely abolished specific migration to CCL19 and CCL21 (data not shown). To further investigate the effects of PGE_2_ and T0901317 on CCR7 expression, we repeated migration assays with freshly isolated human blood monocytes. Results showed that monocyte migration toward CCL19 and CCL21 is increased following treatment of blood monocytes with PGE_2_ and T0901317 (Figure 3).

**Figure 4:**
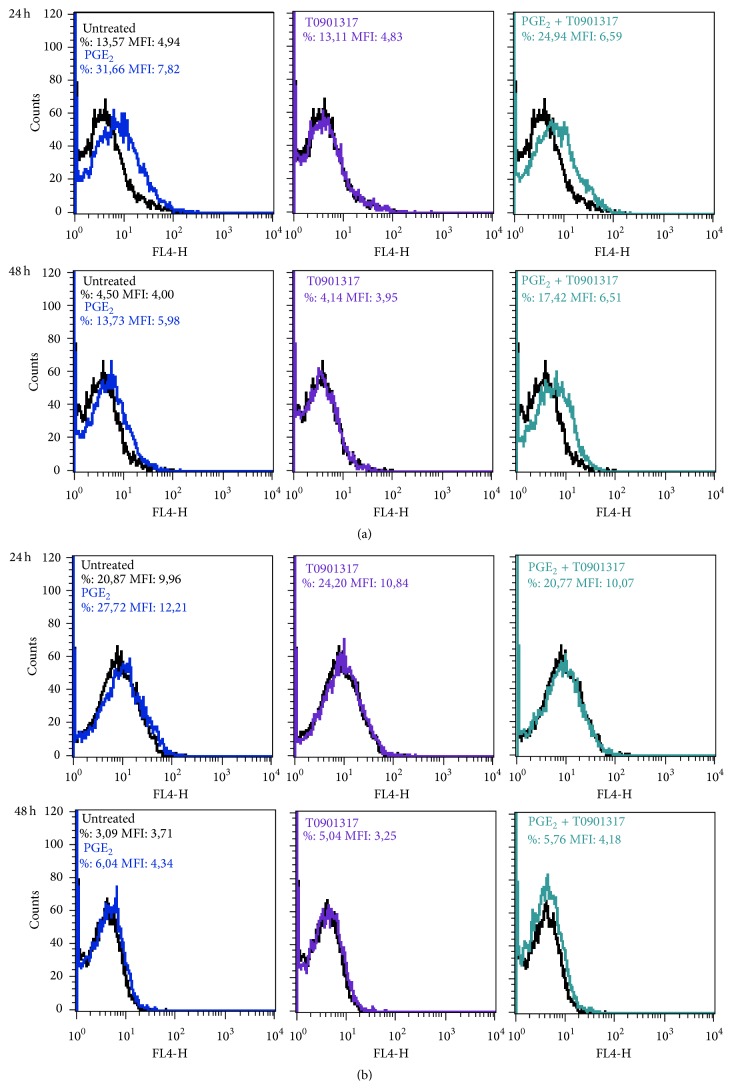
Cell surface and intracellular expression of CCR7 in MM-1 cells. CCR7 surface expression (a) or intracellular expression (b) was evaluated by flow cytometry after treatment with 1 *μ*M PGE_2_ and/or 1 *μ*M T0901317 for 24 h or 48 h. Data shown are from one of three representative experiments.

We aimed at establishing whether the CCR7 receptor is expressed at the cell surface of PGE_2_- and T0901317-stimulated MM-1 cells. Cells were incubated in the presence or absence of PGE_2_ and T0901317 for 24 and 48 h. Surface expression of CCR7 was analyzed using flow cytometry. Our results showed that MM-1 cells basally express CCR7 (13.57% with a MFI (mean fluorescence intensity) of 4.94). However, after 24 h, CCR7 cell surface expression was upregulated by 1 *μ*M PGE_2_ (31.66% with a MFI of 7.82) but not by T0901317 (13.11% with a MFI of 4.83) (Figure 4(a), upper panel). Similar results were observed after 48 h (Figure 4(b), lower panel). Statistics for CCR7 cell surface expression are presented in Table 2. Because CCR7 cell surface expression was not upregulated following the addition of PGE_2_ and T0901317, we performed intracellular flow cytometry assays after 24 h (Figure 4(b), upper panel) and 48 h (Figure 4(b), lower panel) to determine whether CCR7 receptors are trapped internally. We observed that, after 24 h, the basal level of CCR7 is higher than the surface levels (20.87% with a MFI of 9.96), which is similar with PGE_2_ treatment (27.72% with a MFI of 12.21). However, there is no variation between treatments: In addition, there is no variation internal CCR7 expression between MM-1 cells treated with PGE_2_ (24.20% with a MFI of 10.84) or with PGE_2_ in combination with T0901317 (20.77% with a MFI of 10.07). When MM-1 cells are treated for 48 h, decreased levels of CCR7 are observed compared with 24 h treatment: untreated (3.09% with a MFI of 3.71), PGE_2_ (6.04% with a MFI of 3.34), T0901317 (5.04% with a MFI of 3.25), and PGE_2_ in combination with T0901317 (5.76% with a MFI of 4.18).

### 3.2. The EP_2_ and EP_4_ Receptors Are Involved in LXR*α* Activation and PGE_2_-Induced CCR7 Transcription and Functional Migration

**Figure 5:**
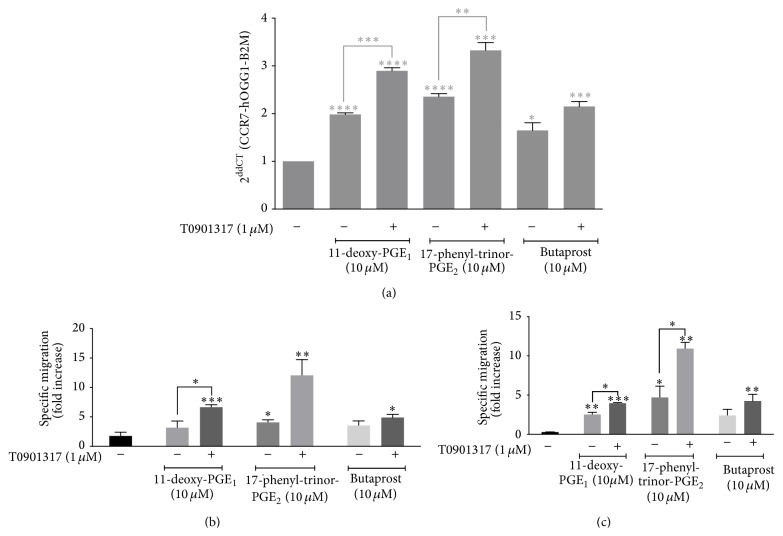
Functional migration in response to PGE_2_ and T0901317 is dependent on EP_2_ and EP_4_ receptors in MM-1 cells. (a) MM-1 cells were treated for 8 h (filled black) and 24 h (filled gray) with the indicated concentrations of 11-deoxy-PGE_1_, 17-phenyl-trinor-PGE_2_, and butaprost. Total RNA was extracted and* CCR7* transcripts were detected using real-time qPCR. Data represent mean ± SD of three independent experiments. Chemotaxis assays in response to 300 ng/mL CCL19 (b) or CCL21 (c) were performed using MM-1 cells stimulated for 48 h with 1 *μ*M T0901317. The mean number of spontaneously migrating cells (that migrated to media alone) was subtracted from the number of cells that migrated in response to CCL19. Data represent mean ± SD of three independent experiments. ^*∗*^ *p* < 0.05, ^*∗∗*^ *p* < 0.01, ^*∗∗∗*^ *p* < 0.001, and ^*∗∗∗∗*^ *p* < 0.0001.

We previously showed that monocytes primarily express two PGE_2_ receptors, EP_2_ and EP_4_ [7]. Moreover, we demonstrated that both receptors are implicated in PGE_2_-induced CCR7 upregulation in MM-1 cells [7]. Thus, using pharmacological agonists for PGE_2_ receptors, we next determined whether EP_2_ and/or EP_4_ play a role in PGE_2_- and T0901317-induced CCR7 migration (Figure 5). Treatment of MM-1 cells with the EP_2_ and EP_4_ agonist 11-deoxy-PGE_1_ increased* CCR7* mRNA levels compared to untreated cells (Figure 5(a)). Although 17-PT-PGE_2_ is described as an EP_1_/EP_3_ (EP_1_ > EP_3_) agonist, at high doses, 17-PT-PGE_2_ also activates EP_4_ receptor (Ki = 1 *μ*M) [22, 23]. Our results showed that 17-PT-PGE_2_ significantly enhanced* CCR7* expression whereas butaprost alone or in combination with T0901317 slightly increased* CCR7* RNA levels compared to control. To determine which PGE_2_ receptors regulate the migratory response of T0901317-treated monocytes to CCL19 and CCL21, chemotaxis assays were performed on MM-1 cells in response to CCL19 (Figure 5(b)) or CCL21 (Figure 5(c)). Our results indicated that MM-1 cells migrated efficiently toward CCL19 or CCL21 when butaprost, 11-deoxy-PGE_1_, and 17-PT-PGE_2_ were added to the milieu. However, the most remarkable migration occurred when PGE_2_-treated MM-1 cells were cultivated in the presence of T0901317.

**Figure 6:**
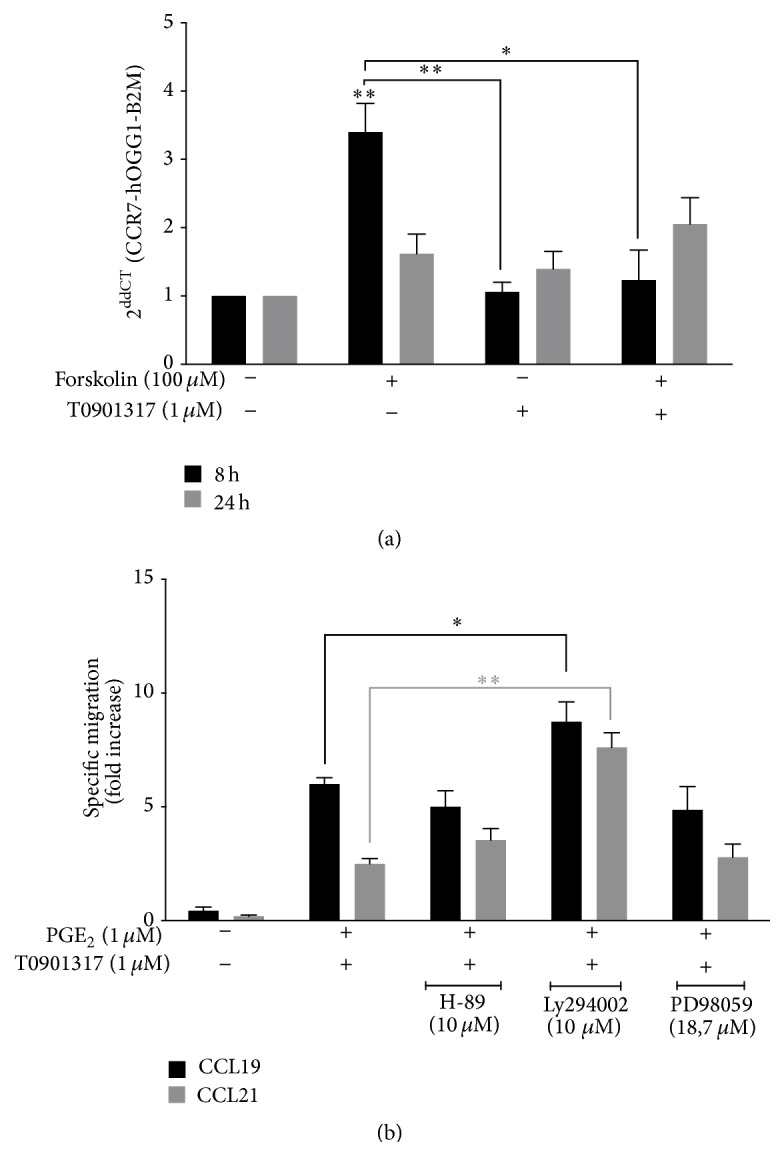
The role of cAMP and alternative EP_2_/EP_4_ signaling pathways in PGE_2_- and T0901317-induced* CCR7* mRNA expression and function. (a) MM-1 cells were treated for 8 h (filled black) and 24 h (filled gray) with 100 *μ*M forskolin alone or in combination with 1 *μ*M T0901317. Total RNA was extracted and* CCR7* transcripts were detected using real-time qPCR. (b) Chemotaxis assays in response to 300 ng/mL CCL19 (filled black) or CCL21 (filled gray) were performed in MM-1 cells treated with the indicated concentrations of H-89, Ly294002, and PD98059 before the addition of 1 *μ*M PGE_2_ and T0901317 for 48 h. The mean number of spontaneously migrating cells (that migrated to media alone) was subtracted from the number of cells that migrated in response to CCL19 or CCL21. Data represent mean ± SD of three independent experiments. ^*∗*^ *p* < 0.05, ^*∗∗*^ *p* < 0.01.

Because EP_2_ and EP_4_ receptors activate adenylate cyclase (AC), which then increases intracellular cAMP levels [23], we next assessed whether forskolin, a pharmacological activator of AC, contributed to the observed induction of* CCR7* mRNA (Figure 6(a)). MM-1 cells were stimulated with 100 *μ*M forskolin for 8 h or 24 h in the presence or absence of T0901317. We found that forskolin alone increases* CCR7* mRNA transcription whereas forskolin combined with T0901317 did not affect* CCR7* expression. AC activation did not modulate* CCR7* mRNA production in T0901317-treated MM-1 cells. Thus, we searched for alternative signaling pathways. MM-1 cells were treated with H-89 (a PKA inhibitor), LY294002 (a PI3K inhibitor), or PD98059 (a MEK inhibitor) before the addition of PGE_2_ and T0901317 for 48 h. Cells were used in migration assays in response to CCL19 or CCL21 (Figure 6(b)). The results showed that selective inhibition of PKA and MEK did not affect CCR7-dependent migration of MM-1 cells in response to CCL19 or 21. In contrast, inhibition of PI3K further enhanced MM-1 cell migration mediated by PGE_2_ and T0901317.

## 4. Discussion

In addition to modulating cholesterol homeostasis, LXRs have emerged as important regulators of inflammatory gene expression and innate immunity [21]. In inflammation, PGE_2_ is of particular interest because its deregulation is associated with the pathogenesis of various diseases and numerous tumor types [11, 24]. In this study, we showed for the first time that PGE_2_ in combination with LXR*α* activation strongly increased the CCR7-dependent migratory capacity of MM-1 cells, a monocytoid cell line. We showed that although* CCR7* mRNA levels are upregulated following treatment with PGE_2_ and T0901317, there was no change in CCR7 cell surface expression. In addition, our results indicate that EP_2_ and EP_4_ receptors are implicated in PGE_2_-mediated* CCR7* transcription required for upregulating the migratory capacity of MM-1 cells in response to CCL19 and CCL21.

In mature DCs, it has been previously shown that activation of LXRs interferes with CCR7 expression, resulting in a dampened antitumor immune response [18]. Recently, Bruckner et al. [19] demonstrated that PGE_2_ rescues the migratory capacity of DCs cultivated in the presence of LXR ligands to migrate toward CCR7 ligands. In contrast to these results, LXR activation in MM-1 cells by the synthetic ligand T0901317 does not modify* CCR7* expression (Figure 1(a)) or CCR7-dependent migration in response to CCL19 (Figure 2(a)) or CCL21 (Figure 2(b)). However, the combination of PGE_2_ and T0901317 significantly increased* CCR7* mRNA production and MM-1 cell migration compared to cells treated with PGE_2_ or T0901317 alone. In addition, blood monocytes treated PGE_2_ and T0901317 migrate in response to CCR7 specific ligands compared to untreated monocytes (Figure 3). Importantly, MM-1 surface expression of CCR7 (Figure 4(a)) did not correlate with migration efficiency (Figure 2). Indeed, other studies have shown that PGE_2_ significantly enhances DC migration through an unknown mechanism that does not depend on the magnitude of CCR7 expression [12, 25, 26]. This phenomenon is also observed with MoDCs matured in the presence of both PGE_2_ and T0901317 [19]. Thus, our results in monocytes are consistent with those observed by other groups in DCs. In addition, flow cytometry analyses were also performed to detect intracellular changes in CCR7 protein expression (Figure 4(b)). However, no changes in expression were detectable. Together, our results demonstrate that LXR activation and PGE_2_ stimulation of MM-1 cells profoundly affect monocyte migration in response to CCL19 and CCL21. Further studies are required to examine the effect of LXR activation in the presence of PGE_2_ on CCR7-dependent migration of freshly isolated blood monocytes from healthy donors.

In macrophages, LXR activation results in the synthesis of the cholesterol efflux transporter ABCG1 as well as of LXR*α* itself through an autoregulatory mechanism [21]. In DCs, PGE_2_ downregulated basal expression of* LXRα* but also inhibited T0901317-mediated autoinduction of* LXRα* [19]. In addition, T0901317-mediated ABCG1 induction was also significantly reduced in MoDCs matured in the presence of PGE_2_ [19]. In our study, we observed that PGE_2_ did not modulate* ABCG1* mRNA production whereas T0901317 alone significantly affected transcription (Figure 1(b)). In contrast, in MM-1 cells stimulated with PGE_2_ and T0901317 for 24 h, we observed significantly reduced* ABCG1* mRNA production. In MM-1 cells, LXR*α* activation upregulated* LXRα* mRNA expression but PGE_2_ had no effect. In contrast to results observed in DCs [19], 24 h treatment with PGE_2_ and T0901317 strongly increased* LXRα* mRNA transcription. Our results demonstrated that the addition of PGE_2_ to T0901317-treated MM-1 cells reduced* ABCG1* mRNA production. Because* LXRα* expression was increased after MM-1 cells were treated with PGE_2_ and T0901317 (Figure 1(c)), the effect on* ABCG1* is likely LXR*α*-independent. It has been shown that ABCG1 and ABCG4 act in concert with ABCA1 to maximize removal of excess cholesterol from cells [27]. Taken together, our results suggest that the presence of PGE_2_ and oxysterols negatively impacts cholesterol efflux in monocytes. Moreover, the presence of these two lipids derivatives may favor intracellular cholesterol accumulation, thereby leading to deregulation of cholesterol homeostasis in monocytes. However, further studies are needed to clarify the mechanisms underlying the observed modulation of* ABCG1* by PGE_2_ and LXR*α* ligands in monocytes.

The EP_2_ and EP_4_ receptors were previously shown to be important for CCR7 expression in mature MoDCs [13, 19, 25] and monocytes [7]. Here, our results showed that PGE_2_ binding to the EP_4_ receptor subtype, and to a lesser extent EP_2_, triggered signals that led to* CCR7* mRNA expression and CCR7-dependent migration of MM-1 cells cultivated in the presence of the LXR*α* synthetic ligand (Figure 5). Interestingly, our data showed that PGE_2_- and T0901317-upregulation of CCR7 expression and function is independent of the cAMP/PKA or MEK branches of EP_2_/EP_4_ signaling (Figure 6). However, we showed that inhibition of the PI3K pathway is responsible for enhanced CCR7-dependent MM-1 cell migration. Thus, our results suggest the contribution of other signaling pathways.

**Figure 7:**
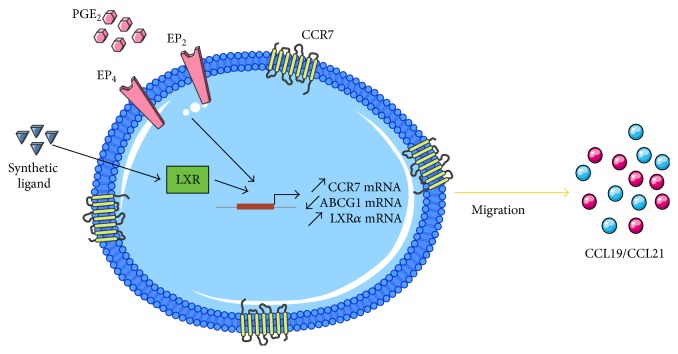
Schematic depicting effects of PGE_2_ and LXR activation on CCR7 functionality in MM-1 cells.

In summary, our results demonstrate that PGE_2_, in combination with LXR*α* activation, increased CCR7-dependent migration of MM-1 cells (Figure 7). Lipid derivatives, including PGE_2_ and oxysterols, may also favor cholesterol accumulation in monocytes. Therefore, our results may have important implications regarding the mechanisms that contribute to atherosclerosis. However, further studies are needed to better understand the role of lipid derivatives produced during inflammation.

## Figures and Tables

**Table 1:** Primers used in real-time qPCR assays.

Target	MgCl_2_	Forward primer	Reverse primer
LXR	3.5 mM	5′-GCTGCAAGTGGAATTCATCAACC-3′	5′-ATATGTGTGCTGCAGCCTCTCCA-3′
CCR7	2 mM	5′-GTGGTGGCTCTCCTTGTCAT-3′	5′-TGTGGTGTTGTCTCCGATGT-3′
ABCG1	2 mM	5′-CACCAGCCGACTGTTCTGCAT-3′	5′-TGTACCGGGGAAAAGTCTGC-3′
B2M	2 mM	5′-GAGTATGCCTGCCGTGTGAA-3′	5′-TGCGGCATCTTCAAACCTCC-3′
hOGG1	3.5 mM	5′-TGGAAGAACAGGGCGGGCTA-3′	5′-ATGGACATCCACGGGCACAG-3′

**Table 2:** Statistical analyses of CCR7 surface expression in MM-1 cells.

	Mean % of positive cells	SD	*p* value	Mean MFI	SD	*p* value
24 h						
Untreated	16,04	2,21		4,32	0,54	
PGE_2_	36,40	5,67	0.0044	9,24	1,55	0.0066
T0901317	11,32	1,86	0.047	4,76	0,72	0.45
PGE_2_ + T0901317	26,83	2,06	0.0038	6,68	1,18	0.035
48 h						
Untreated	6,11	2,04		3,32	0,76	
PGE_2_	17,81	3,99	0.011	7,59	2,13	0.030
T0901317	5,07	2,49	0.61	3,71	0,88	0.57
PGE_2_ + T0901317	20,11	2,48	0.0016	8,06	1,69	0.011

Data represent mean ± standard deviation (SD) of three independent flow cytometry experiments after 24 or 48 h of treatment with 1 *μ*M PGE_2_ and/or 1 *μ*M T0901317.
